# Recurrent Pleural Effusions in an Elderly Female Patient With Renal Amyloidosis

**DOI:** 10.7759/cureus.93338

**Published:** 2025-09-27

**Authors:** Ajith S Bharadwaj, Karson Munson, Moneeb Mustafa, Prathapraju Poloju

**Affiliations:** 1 Internal Medicine, Edward Via College of Osteopathic Medicine, Alexandria, USA; 2 Internal Medicine, Edward Via College of Osteopathic Medicine - Louisiana, Monroe, USA; 3 Internal Medicine, CHRISTUS St. Frances Cabrini Hospital, Alexandria, USA; 4 Internal Medicine, Rapid Regional Medical Center, Alexandria, USA

**Keywords:** adult pulmonology, elderly population, general internal medicine, general nephrology, rare cause of pleural effusion, renal pathology, sytemic amyloidosis

## Abstract

Amyloidosis is a rare and severe disease process that is often systemic in nature. Clinical presentations of patients include involvement of various organ systems such as the kidneys, heart, and lungs. As such, presentations of amyloidosis can be peculiar and can greatly mimic other conditions, making diagnosis very difficult. Delays in diagnosis or misdiagnoses can lead to significant morbidity and great potential for multi-organ dysfunctions.

We describe the case of an elderly female patient without a known cardiac history who presented to the emergency department with increasing shortness of breath. This patient had a recent history of renal amyloidosis requiring regular chemotherapy. The patient was found to have extensive secondary fluid buildup in the pleural space, resulting in recurrent pleural effusions requiring repeated thoracenteses. Pleural effusions are rare complications of systemic amyloidosis that are poorly understood. After thorough evaluation of the cardiac and renal systems and ruling out malignancy, we suspected that direct infiltration of the pleura with amyloid fibrils and exacerbation of fluid overload from renal failure led to the disease presentation. With proper management of the patient’s end-stage renal disease with dialysis, the fluid-overload state with loop diuretics, and effective use of thoracentesis, the patient’s condition showed improvement. This report serves to educate on the pathophysiology of amyloidosis and provide a clinical anecdote of the presentation, course of this chronic disease process, treatment methodologies, and future of therapy.

## Introduction

Amyloidosis describes a group of diseases characterized by extracellular deposits of beta-sheet fibrils, an aggregation of amyloid proteins. As of today, there are at least 42 proteins known to be capable of amyloid fibril formation [1]. Many of these proteins are normal constituents of plasma. The process of fibril formation involves insoluble subunit polymers forming from the misfolding or unfolding of soluble subunit polymers. The insoluble subunit polymers undergo conformational changes that lead to the formation of a predominantly antiparallel beta-pleated sheet. These sheets then auto-aggregate and come together to form beta-sheet fibrils. The misfolding or unfolding of soluble subunit polymers may be caused by acidification or proteolysis and accelerated by nucleation. Additionally, the deposition of other non-fibrillar substances, such as glycosaminoglycans (particularly heparan sulfate), serum amyloid P component (SAP; a pentraxin family member that also includes C-reactive protein), and apolipoprotein E, may play an important role at various stages of fibrillogenesis. It may also influence the deposition in tissues and influence resorption. These fibrils can deposit anywhere in the body, including the lungs, heart, gastrointestinal tract, muscles, joints, and skin. Some are deposited at the site of amyloid production (localized amyloid), while some circulate in the blood to deposit in different organs and tissues (systemic amyloid). There are several different types of localized and systemic amyloidosis, and while the pathophysiology of amyloidosis remains the same, the cause of the misfolding depends on the specific type. Some types of amyloidosis are clearly hereditary, resulting from mutations in precursor proteins that cause either missense, deletion, or premature stop codon mutations. There are 28 localized forms of amyloidosis and 18 systemic forms; however, three forms predominate [1]. The three predominant forms include:

 1. AL amyloidosis, caused by a plasma cell dyscrasia, which leads to protein deposition derived from light chain fragments.

 2. ATTR amyloidosis, which may occur as a wild-type form associated with aging, or as mutant proteins associated with familial neuropathy or cardiomyopathy.

 3. AA amyloidosis, which may occur as a potential byproduct of chronic inflammatory conditions that result in a sustained increase in serum amyloid A protein, an acute phase reactant, which can aggregate and form amyloid deposits.

As stated before, amyloidosis refers to a group of disorders characterized by the result of extracellular deposition of misfolded protein fibrils, which damage normal tissue structure and function. Among the systemic forms, AL amyloidosis is the most common form and has documented sequelae. The prevalence of the AL subtype is estimated to be 10 cases per million people per year, and renal involvement as a complication is seen in approximately 50% to 80% of patients with AL amyloidosis [2,3]. Persistent pleural effusions develop in 1% to 2% of patients with AL amyloidosis, making it a rare complication of this disease process [4].

Renal amyloidosis describes the disease process that is the result of the accumulation of beta-sheet fibrils in the kidneys. The clinical manifestations of renal amyloidosis depend on the type of amyloid protein, the specific location within the kidneys, and the severity of amyloid deposition. For example, in patients who have significant tubular amyloid deposits, syndromes that represent tubular dysfunction, such as renal tubular acidosis, acquired Fanconi syndrome, or arginine vasopressin resistance, can occur [2]. AL renal amyloidosis often presents with nephrotic-range proteinuria, hypoalbuminemia, and progressive chronic kidney disease [4]. It has been estimated that approximately 75% of patients with AL amyloidosis present with proteinuria, making it the most common manifestation seen with amyloid deposition in the glomerulus [2,4]. The degree of proteinuria can range from mild to massive, depending on the degree of glomerular involvement. End-stage renal disease (ESRD) develops in approximately 20% of amyloidosis patients presenting with nephrotic syndrome [2]. This proteinuria and renal dysfunction are often accompanied by edema, which can lead to volume overload and the development of transudative pleural effusions, contributing to symptoms such as shortness of breath, as seen in this patient. It is also thought that amyloid fibrils may even infiltrate the pleura, inducing leakage of fluid into the pleural space and impairing drainage from the pleural cavity, exacerbating fluid accumulation [3].

Amyloidosis can be a great mimicker in medicine. It can affect any organ system and cause a wide variety of symptoms. Therefore, it can be extremely difficult to diagnose, as other diagnoses are often favored initially. For instance, with this patient, the most likely diagnosis would be heart failure secondary to cardiorenal syndrome. However, this patient’s unremarkable cardiac work-up ruled out that diagnosis and thus required a deeper search into the etiology of this patient’s symptoms. This case report is paramount in highlighting the importance of maintaining a high index of suspicion, particularly when common presentations occur in patients with a known systemic disease such as AL amyloidosis.

## Case presentation

A 71-year-old female with a history of biopsy-proven renal amyloidosis diagnosed four months before the current presentation and chronic kidney disease (CKD), arrived at the emergency department with dyspnea that began two weeks ago. The patient denied having chest pain, edema, or weight gain. She was admitted to another hospital at the time and was finishing treatment for diarrhea caused by Clostridioides difficile. Nearing the end of that prior hospitalization, she began to experience SOB, for which she underwent bilateral thoracentesis to remove 1 L of pleural fluid from each side. The procedure had caused a rapid improvement in her symptoms, and it was thought that her symptoms had resolved. However, within the week before current admission, she noted significantly worsening shortness of breath (SOB) like before, necessitating her arrival at the emergency department.

Further investigation revealed that the patient had missed the last two appointments of chemotherapy for the treatment of her amyloidosis due to inclement weather and her recent hospitalization. At the time of presentation, history was not available on the characteristics of the pleural fluid extracted at prior admission, nor on the exact subtype of the amyloidosis (AL, AA) that the patient had.

At the emergency department, the patient was evaluated thoroughly to further investigate the cause of her presentation. Assessment of her vitals revealed a body temperature of 97.8 °F, heart rate of 83 beats per minute, and a blood pressure of 157/80 mmHg. Her pulse oxygenation stat (O_2_ stat) was 99% on a 2 L/min nasal cannula with a respiratory rate of 18 cycles/minute. Physical exam revealed a frail appearing elderly woman who was short of breath, noted by accessory muscle use despite her O_2_ stat. There was also significant pitting edema seen in her lower extremities. No other relevant findings were found.

**Figure 1:**
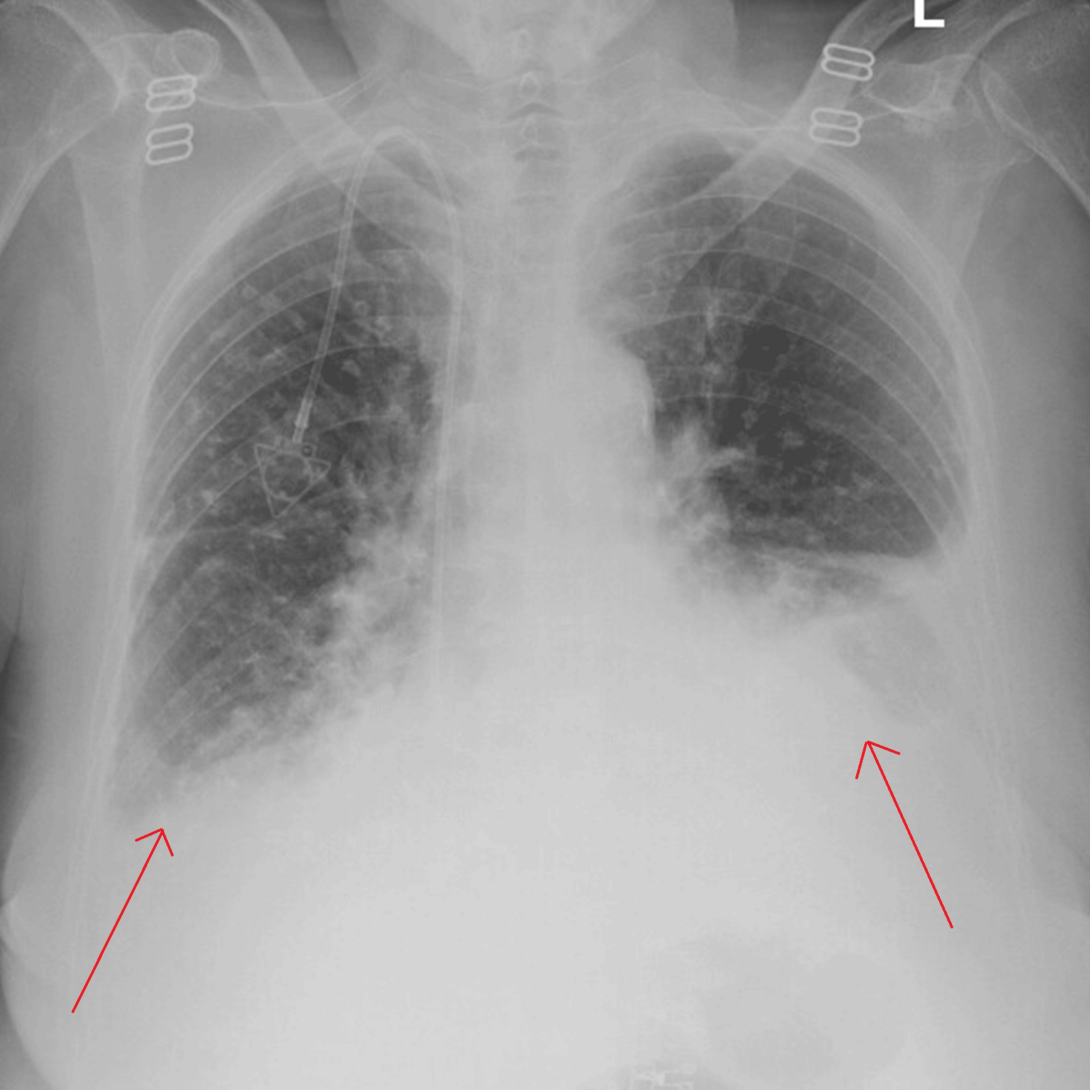
Chest X-ray revealing significant bilateral costophrenic angle blunting (red arrows) and scattered pulmonary nodules.

To further evaluate the patient, a complete blood count with differential (CBC w/diff), a complete metabolic panel (CMP), an antero-posterior chest radiograph (AP CXR), and thoracentesis were ordered. Pertinent lab findings from the CBC w/diff and CMP include a hemoglobin and hematocrit level of 8.2 g/dL and 26.9%, a hemolyzed potassium level of 5.5 mmol/L, a creatinine level of 4.66 mg/dL, and an estimated glomerular filtration rate (eGFR) value of 9 mL/minute/1.73 m^2^. High sensitivity troponin levels were measured at 43 ng/L on admission. Imaging revealed multiple small nodules scattered across the right upper lobe and left upper lobe of the lungs. Most prominently, pleural effusions were noted with atelectatic changes at the left base of the lung, consistent with the history of prior pleural effusions and presentation of SOB. Figure 1 showcases these initial AP CXR findings.

Additionally, due to a strong recent history of recurrent bilateral effusions, thoracentesis was performed to evaluate the cytochemical characteristics of the pleural fluid. Table 1 highlights the cytology and pleural fluid analysis. A total volume of 1,450 mL from the left and 1,260 mL from the right hemithorax was extracted during the procedure. Pertinent cytology findings revealed reactive mesothelial cells, histiocytes, and lymphocytes in the absence of malignant cells [5]. A cardiac work-up, including electrocardiography (ECG) and echocardiography, was unremarkable.

**Table 1:** Thoracentesis fluid analysis and cytology.

Test	Result	Flag	Reference
Fluid color	Straw	L	Yellow
Fluid appearance	Clear		Clear
Fluid source	Thoracic fluid		
Nucleated cells, field	248		
Red blood cells	113		
% Segmented neutrophils	17		%
% Lymphocytes	38		%
% Monocytes	15		%
% Eosinophils	5		%
Macrophages, field	17		
Mesothelial, field	8		
Culture, body fluid			
Smear, Gram stain	Many white blood cells were seen; no organisms were seen.		
Culture, body fluid final	No growth		
Culture, anaerobic final	No anaerobes cultured		

Based on the clinical history, examination, labs, and imaging, and thorough consideration of differentials, a diagnosis of pleural effusions secondary to systemic renal amyloidosis was made. Inpatient management was based on addressing individual system dysfunctions. The treatment plan contained multiple stages and was multidisciplinary in nature to properly address patient symptoms and concerns. Multidisciplinary expertise and coordination enabled the highest level of optimization of treatment and continuity of care. The first entailed getting a nephrology consult to start dialysis to optimally treat the patient's CKD and further declining renal function. Based on recommendations by nephrology, the patient was started on fluid restrictions, bumetanide 0.5 mg IV daily to treat the fluid overload state, and avoidance of non-steroidal anti-inflammatory drugs (NSAIDs) and other nephrotoxic medications. The second stage of the treatment plan involved getting a consultation with cardiothoracic surgery for thoracentesis and to discuss potential treatment options such as pleurodesis and an indwelling pleural catheter (IPC) as solutions for addressing the recurrent pleural effusions. With this treatment plan, we saw the patient's labs, SOB, and lower extremity pitting edema improve over the next week, and ultimately she was discharged for treatment in the outpatient setting. Finally, the third step involved outpatient follow-up and management to address the root cause: amyloidosis. This involved treatment of amyloidosis with regular chemotherapy and renal function monitoring, outpatient with the patient's preferred primary care provider. Before being discharged, our patient was educated on the importance of attending regular chemotherapy treatment sessions for amyloidosis and dialysis for her CKD to ensure a decreased risk of exacerbations in the future. It is expected that this effective outpatient regimen will enable our patient to experience a better quality of life through the reduction of amyloid fibril deposition and fluid overload, aiding in the reduction of symptomatology.

## Discussion

Amyloidosis often presents with multi-system involvement and can closely mimic a variety of other conditions. As such, it is exceedingly important to have a refined and pertinent list of differential diagnoses. In the evaluation of our patient, heart failure and malignancy with metastasis were two major considerations to explain the etiology of the pleural effusion.

The first and most important differential as part of the list was heart failure. Given the patient’s history of renal amyloidosis, it is important to consider the possibility that amyloid proteins may have deposited onto cardiac tissues, resulting in restrictive cardiomyopathy and heart failure [6]. Previous studies have shown that symptoms of amyloidosis may include increasing SOB, pleural effusions, and other symptoms of heart failure, similar to our patient's presentation [7]. Heart failure secondary to cardiorenal syndrome and CKD was highly considered, given how ESRD can impact heart physiology and function. Our patient presented with severely diminished kidney function secondary to amyloidosis, which increased the likelihood that this was the likely cause of the effusions. Analyzing the CMP over several days revealed a baseline eGFR value that ranged between 9 and 12 mL/minute/1.73 m^2^. Based on these findings, kidney staging indicated that our patient had Stage 5 chronic kidney disease and, consequently, required hemodialysis during hospitalization, with outpatient continuity of care after discharge [8]. To further establish a relationship between renal failure and heart failure, a combination of labs such as brain natriuretic peptide (BNP), CXR, ECG, echocardiography, and high-sensitivity troponin was collected. However, they were relatively unremarkable, except for troponin. Although elevated high-sensitivity troponin levels would normally indicate heart pathology, it is known that in cases of end-stage CKD, as with our patient, these values may be elevated due to decreased clearance [9]. Given the absence of a cardiac cause in the development of the effusions, renal failure caused by amyloidosis and fibril-mediated damage of the pleura was considered to be the most probable pathology contributing to our patient's condition.

The second major differential diagnosis we considered in our patient was malignancy with metastasis to the lungs. In our patient, CXR demonstrated the presence of nodules in the right and left upper lobes of the lung as well as perihilar effusion/nodules. Given the rapid onset of pleural effusion and recurrence, it was important to have a high degree of clinical suspicion for malignancies. Tests to rule out malignancies and metastasis include thoracentesis and fluorodeoxyglucose positron emission tomography and computed tomography (F-FDG PET/CT) scans. In the context of the history and presentation, we were concerned for malignant pleural effusions caused by neoplasia such as mesothelioma and the metastasis of various cancers [10]. Thoracentesis was planned to be the initial diagnostic step. If this were to be positive, a further workup may have been warranted with the F-FDG PET/CT scan [11]. However, in our case, a F-FDG PET/CT scan was not necessary as the pleural fluid cytology was negative for malignant cells. Furthermore, the cytology report of the fluid revealed a pattern of reactive mesothelial cells, histiocytes, and lymphocytes similar to other reported cases of amyloidosis, further providing evidence in support of pleural effusions secondary to amyloidosis [5].

Having ruled out cardiac involvement and malignancy as potential causes of the effusions, we further investigated renal amyloidosis as the primary cause. It is worth noting that our patient's medical records did not indicate what specific subtype of amyloidosis she had. As iterated before, AL amyloidosis is the most common type of amyloidosis that can be encountered, and the cytologic pattern of the pleural fluid and nodular densities on the AP CXR lead us to further suspect systemic AL amyloidosis as the likely cause [12]. Interestingly, AL amyloidosis has been observed to have a co-occurrence with multiple myeloma, although the evidence is not conclusive [13]. In our patient, multiple myeloma was diagnosed a few months post-discharge from this hospitalization, providing additional evidence that our patient's amyloidosis may have been of the AL subtype and systemic. The pathology of pleural effusions in amyloidosis is still not fully understood. However, as mentioned earlier, it is speculated that in AL amyloidosis, direct infiltration and deposition of AL Amyloid fibrils onto the parietal layer of pleura can disrupt the normal physiology of the pleural space, contributing to effusions that can be seen on imaging [14]. With this possibility in mind, we further suspected that the combination of direct pathologic infiltration of pleura by AL fibrils and Stage 5 CKD may have synergistically played a role in the development of the recurrent pleural effusions we saw in our patient.

Specific therapy for amyloidosis depends on the type. To best treat patients with amyloidosis, the diagnosis must be confirmed. In addition, the evaluation must establish the location, extent of involvement, and comorbidities that may affect the treatment options. Initial laboratory studies should include a CBC w/diff, chemistries with renal and hepatic function, an electrolyte panel, electrophoresis of the serum and urine, immunofixation electrophoresis of the serum and urine, 24-hour urinary protein and creatinine clearance, serum free light chain assay, troponin T and troponin I, BNP, thyroid stimulating hormone (TSH), prothrombin time, and partial thromboplastin time. Also, a unilateral bone marrow aspirate and tissue biopsy with immunohistochemical staining or flow cytometry for kappa and lambda and Congo red staining for amyloid should be performed. A direct organ biopsy is not required if there is evidence of organ involvement with a previous confirmation of an amyloidosis diagnosis at another site [15]. 

To diagnose renal amyloidosis specifically, a direct biopsy revealing amyloidosis with evidence of organ dysfunction or a 24-hour urine protein greater than 0.5 g/day with a predominance of albumin, with other causes of proteinuria excluded, serves as confirmation [15]. The markers and thresholds used to stage renal amyloidosis include an eGFR of less than 50 mL/minute/1.73 m^2^ and a proteinuria measurement greater than 5 g/24 hours. Stage I renal amyloidosis is when the eGFR is above the cutoff and the proteinuria is below the cutoff. Stage II renal amyloidosis occurs when either the eGFR is below the cutoff or the proteinuria is above the cutoff. Lastly, stage III renal amyloidosis is when both the eGFR is below the cutoff and the proteinuria is above the cutoff [16].

Renal amyloidosis requires urgent treatment to prevent the progression of kidney decline to ESRD. Treatment involves targeting the production of the amyloid protein, in addition to supportive measures to treat secondary manifestations like pleural effusions. The goal is to achieve the best possible reduction in the amyloid precursor to limit further kidney injury and preserve kidney function. For patients with stage I to IIIA AL amyloidosis, the recommendation is to begin a chemotherapy regimen of daratumumab, cyclophosphamide, bortezomib, and dexamethasone (Dara-CyBorD) in 28-day cycles [15]. Daratumumab can be taken as a single agent or in combination with cyclophosphamide and dexamethasone if the patient is not a candidate for bortezomib due to sensory neuropathy that is painful or self-limiting. Following this, further management depends on the patient’s ability to take high-dose (200 mg/m^2^) melphalan followed by autologous hematopoietic cell transplantation (HCT). Those who choose to delay HCT may continue the chemotherapy induction regimen for a total of cycles or months, similar to the regimen for those ineligible for HCT [15]. In the case of our patient, she is ineligible for HCT given the refractory nature of her pleural effusions [3]. Due to the ineligibility for HCT, management of our patient’s renal amyloidosis was complicated by both her age and frailty, necessitating a delicate approach. Although she was undergoing chemotherapy before admission, recent interruptions due to hospitalization and inclement weather led to disease progression. For this patient, chemotherapy remains the primary means of controlling amyloidosis progression and for the management of symptoms. This changed upon admission when the clinical priority became supportive symptomatic therapy. Over the course of hospitalization, this included management of the patient’s fluid-overloaded state and recurrent pleural effusions, which were the cause of her respiratory distress. Upon discharge, chemotherapy became the mainstay of therapy, as is the standard of treatment for this condition with refractory sequelae. As with other systemic conditions, the prognosis of AL amyloidosis is highly variable, dependent on a multitude of factors, including the number, nature, and extent of organ involvement [15].

It is also important to consider barriers to treatment in patients. While educating the patient on the importance of attending regular treatment sessions is vital, it may not be practical to assume that the patient will be able to attend all of the sessions. Barriers such as finances, transportation, or weather can prevent the patient from attending the sessions, which can subsequently lead to complications such as fluid buildup and pleural effusions as seen in this patient. We encourage open dialogue with patients on how to best address their needs and reiterate the importance of a multidisciplinary team in the management of complex disease processes like amyloidosis.

This case reflects the importance of using a standardized and thorough evaluation in a multi-system fashion. It is necessary to understand that with any type of amyloidosis, a manifestation of progressive disease that involves multiple systems is highly probable. In the case of our patient, the renal and pulmonary systems were vastly affected, and with the presence of pitting edema in the lower extremities, it is expected that vascular and cardiac compromise will likely occur as this disease progresses. Therefore, when there is multi-system involvement in the absence of sepsis or major infection, it is important to have a high degree of suspicion for amyloidosis. In such scenarios, including amyloidosis as part of the differential diagnoses will be essential for further investigation. It is also crucial to investigate a strong, pertinent list of differential diagnoses for further work-up. It is also equally important to follow a multidisciplinary plan that involves various specialists to address the problems that can arise from this pathology. This is critical in ensuring prompt treatment of the condition, which is essential for better clinical outcomes. In cases of AL amyloidosis, which is likely present in our patient, when untreated, it can reduce the life expectancy of affected individuals to one year [17]. However, results have shown that with the detection of amyloidosis at earlier stages of disease, prognosis and survivability significantly improve. One study by Barret et al. [18] demonstrated that in stage I of disease, mean survivability was 118 months. With increasing stages, survivability decreased significantly [18]. Reputable institutions such as Cedars-Sinai [19] have published that with adequate therapy, amyloidosis can enter remission, enabling patients to have near-normal life expectancies. In geriatric patients with systemic amyloidosis, like in our case, we also stress the importance of acknowledging the age, disease prognosis, and functional status of patients. Aggressive interventions may not always be appropriate or beneficial. Instead, coordinating management to balance disease control with the patient’s comfort and independence is crucial. Given our patient’s age and functional status, minimally invasive but effective interventions were initially prioritized during hospitalization. She was started on intravenous loop diuretics and underwent therapeutic thoracentesis to relieve the symptoms caused by the pleural effusions. Given the likelihood of recurrence, palliative options such as pleurodesis and IPC placement were also discussed to provide long-term symptom control. For similar reasons, chemotherapy was also recommended to our patient therapeutically for the prevention of exacerbations. Although it is difficult to assess the prognosis of our patient, we believe that these interventions can help our patient maximize the benefit-to-risk ratio relative to her state of health. Furthermore, we encourage open discussions about goals of care with patients to empower their autonomy in decision-making. Involving patients in their care can aid them in better understanding the management of their chronic disease, facilitating clinicians in delivering greater patient satisfaction.

In the future, it is anticipated that new immune therapies may improve the efficacy of treating amyloidosis and its sequelae [20]. As of 2023, clinical trials to assess the effectiveness of immune antibody therapies against deposited amyloid fibrils are ongoing. Studies are investigating immune therapies such as the anti-amyloid monoclonal antibody CAEL 101 and humanized monoclonal antibody Birtamimab in the targeting and clearing of amyloid proteins [3]. If such novel therapies are found to be effective, this may significantly transform the current setting for the treatment of amyloidosis, helping gear towards specialized directed therapy, enabling clinicians to deliver better outcomes in the frail and elderly population [3].

## Conclusions

Renal amyloidosis and systemic amyloidosis can lead to significant morbidity by affecting multiple bodily systems. Amyloidosis can be a great mimicker, presenting in peculiar ways, as seen with the recurrent pleural effusions in our patient. Our case highlights the importance of thorough investigation with refined and pertinent differential diagnoses, early recognition, treatment, and a comprehensive multidisciplinary team-based approach to management. Prompt initiation of dialysis, targeted fluid management, and procedural interventions such as thoracentesis played key roles in stabilizing our patient's condition and improving her overall trajectory. We also stress the importance of open discussion with patients regarding goals of care, given the chronicity of the disease process, and addressing barriers to treatment. This case reinforces that in rare conditions like amyloidosis, a thorough workup and high degree of clinical suspicion in conjunction with proactive and coordinated care are essential to preserving organ function, reducing hospitalizations, and ultimately improving patient outcomes.
